# Partial nephrectomy for solid renal masses in polycystic kidneys: open surgical technique

**DOI:** 10.1308/rcsann.2025.0052

**Published:** 2025-10-01

**Authors:** C O’Connell, MR Clarkson, F O’Brien, P Russo

**Affiliations:** ^1^ Cork University Hospital, Ireland; ^2^ Memorial Sloan Kettering Cancer Centre, New York, USA

**Keywords:** Kidney cancer, Polycystic kidney, Partial nephrectomy, Open surgery

## Abstract

Partial nephrectomy for small renal masses is well established as the preferred means of surgical treatment for kidney cancer, to preserve renal function. Although minimally invasive techniques are now standard in many countries, open surgery remains an important technique for difficult cases. Partial nephrectomy in patients with autosomal dominant polycystic kidney disease (ADPKD) is challenging and not widely reported. We describe the open surgical technique for partial nephrectomy in patients with multicystic kidneys who are not on renal replacement therapy (RRT). We illustrate the technique using three cases of patients with multicystic kidneys and solid renal masses. All had early chronic kidney disease, making partial nephrectomy for suspected kidney cancer the preferred strategy. The patients had stable creatinine and were recurrence-free at last follow-up. Open partial nephrectomy remains an important surgical technique for resection of challenging kidney tumours, such as those in multicystic kidneys. Patients with ADPKD and solid renal masses who are not on RRT should be managed in the same manner as the background population, with nephron-sparing surgery wherever possible.

## Background

Partial nephrectomy for solid renal masses is the preferred treatment modality for patients with autosomal dominant polycystic kidney disease (ADPKD) who are not on renal replacement therapy (RRT), because nephron-sparing surgery may delay the need for dialysis or renal transplant. A nephron-sparing approach in this cohort presents a challenge, because of innumerable renal cortical cysts and abnormal renal architecture, making both correct identification of the mass in question and post-excision reconstruction more challenging.

ADPKD is the most common inherited form of chronic kidney disease (CKD), and is characterised by progressive renal cyst accumulation and enlargement throughout life.^1^ Secondary complications include intracyst haemorrhage, pain from ruptured cysts, cyst infection, abdominal distension owing to renal enlargement, and progressive kidney failure, with most patients progressing to end-stage renal disease requiring RRT by the age of 60.^2^ ADPKD accounts for approximately 5% of cases of end-stage renal disease in the US and Europe.^3,^^4^ The true incidence of renal cell carcinoma in patients with ADPKD is not known, but some small series suggest an increased prevalence in this cohort, with one series reporting a 5% prevalence of malignancy in nephrectomy specimens from patients with ADPKD.^5–^^7^ A population study in Taiwan found a significantly increased risk of several solid organ malignancies, including renal cell carcinoma, in patients with ADPKD, with a incidence of 20.1 cancers per 1,000 person years compared with 10.9 cancers per 1,000 person years for age-matched controls.^8^ Increased activation of the mammalian target of rapamycin pathway has been proposed as a potential mechanism for an increase in the risk of neoplasia in polycystic kidneys.^9,10^

Nephrectomy is commonly performed in patients with ADPKD to make space for a kidney transplant; however, partial nephrectomy in this cohort is described in only a small number of cases worldwide. In an extensive literature search, we identified only nine cases of partial nephrectomy in polycystic kidneys. Three cases were reported as far back as the 1950s, for reasons other than renal masses (infection, intractable haematuria).^11–13^ Karnik *et al* described the technical challenges in performing laparoscopic hand-assisted partial nephrectomy in this cohort, reporting increased warm ischaemic time and suggesting that open partial nephrectomy might be a more suitable approach.^14^ Peña *et al* reported a laparoscopic partial nephrectomy for a renal mass in ADPKD without clamping the renal artery, thereby avoiding warm ischaemic time.^15^ One case of bilateral partial nephrectomy for bilateral renal cell carcinomas has been described in a patient with ADPKD.^16^ More recently, two cases of robotic partial nephrectomy were reported, with the estimated glomerular filtration rate (eGFR) unchanged postoperatively in both patients.^17^ In paediatric surgery, there has been one reported case of a partial nephrectomy for excision of Wilms tumour in a 17-month-old child with polycystic kidneys.^18^

We describe the open surgical technique for partial nephrectomy in polycystic kidneys through the illustration of three cases all of whom had early CKD, making nephron-sparing surgery a priority. This study was carried out under the Code of Ethics of the World Medical Association (Declaration of Helsinki). Approval for this case series was granted by the Ethics Committee/IRB of University College Cork and Memorial Sloan Kettering Cancer Center. Patients gave their consent for anonymised clinical images and case descriptions to be published in a scientific journal.

## Case histories

### Case 1

**Figure 1:**
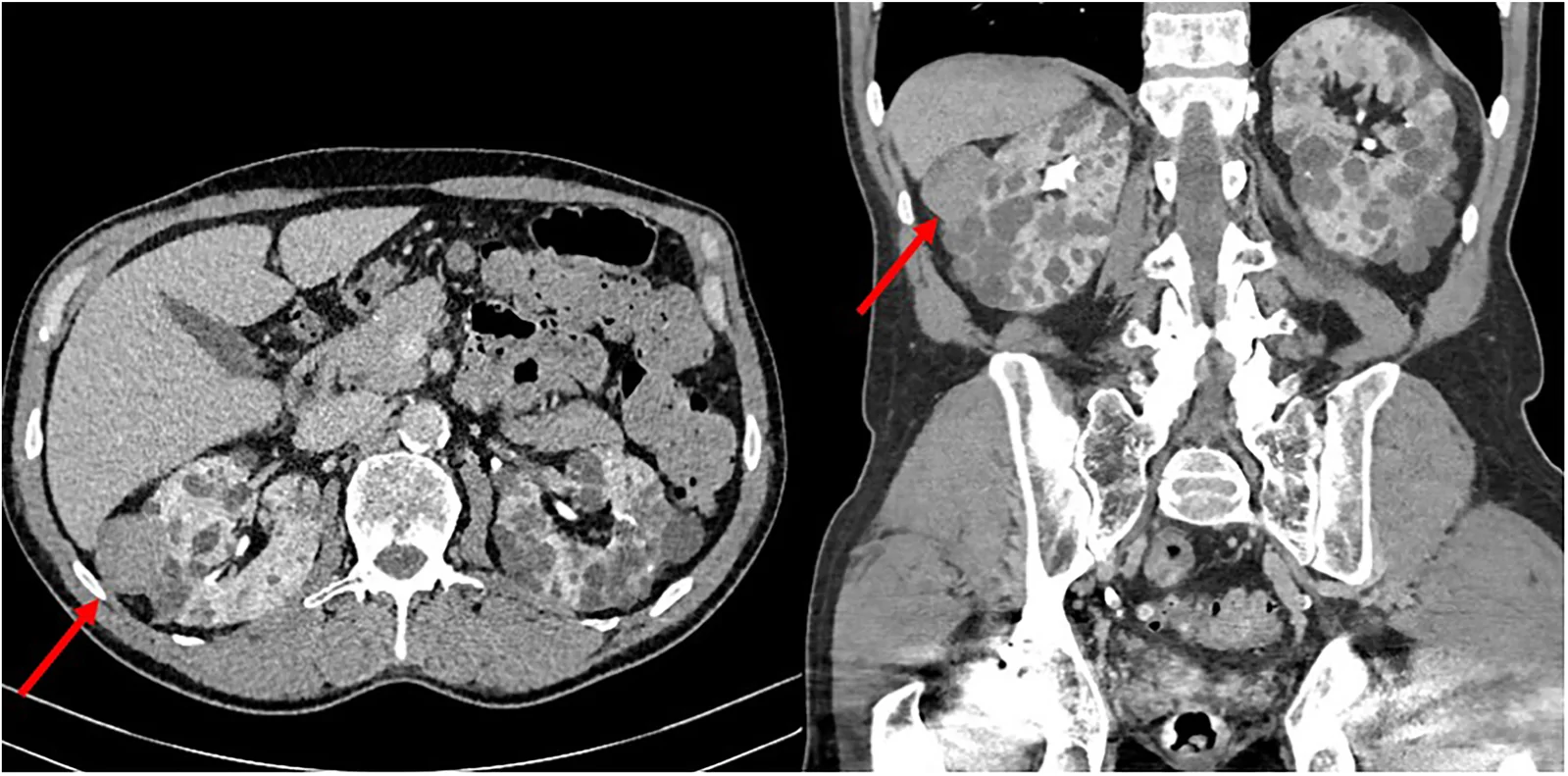
Case 1. Axial and coronal computed tomography images of the 4cm exophytic right renal mass in a patient with autosomal dominant polycystic kidney disease

**Figure 2:**
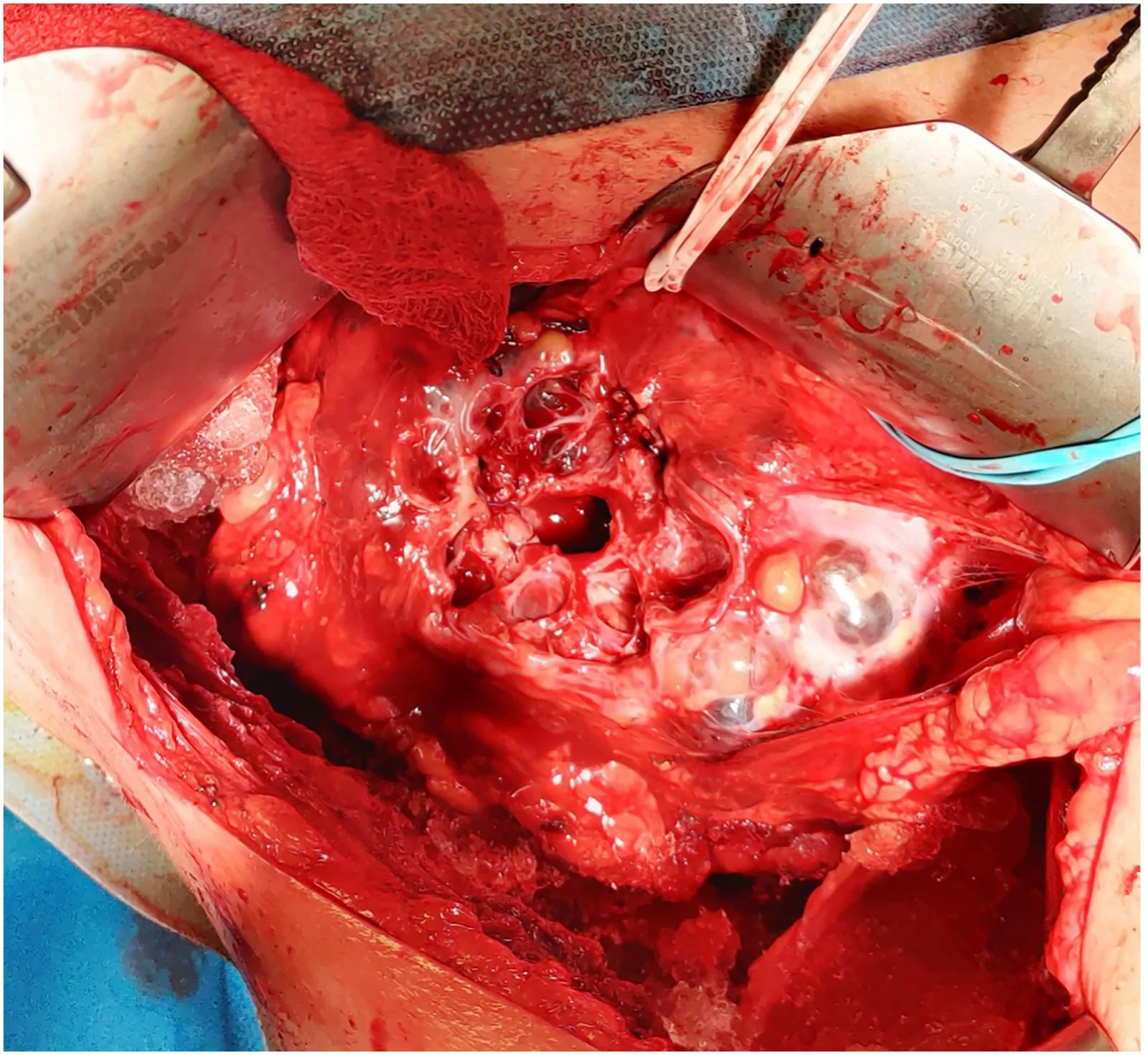
Case 1. Post-excision defect in the renal parenchyma prior to closure

A 67-year-old man with hypertension, stage 2 CKD and radiological evidence of polycystic kidneys presented with an enlarging solid renal mass in the interpolar region of his right kidney (Figure 1). He had a history of superficial urothelial cancer on surveillance, and the renal mass was detected incidentally on upper tract computed tomography surveillance. Baseline creatinine was 100μmol/L, giving an eGFR of 60ml/min. The RENAL nephrometry score for the mass was 6, denoting low complexity.^19^ This reveals a potential pitfall with the RENAL scoring system, because it does not take into account masses in kidneys with innumerable cysts. The mass was approached via a right flank incision. Intraoperative ultrasound confirmed the location of the tumour. The perirenal space was filled with ice slush, bulldog clamps were applied to the renal artery, and the mass was excised with an ischaemic time of 21min, with minimal decortication of surrounding cysts. Renorrhaphy was achieved with Floseal Max and a Surgicel bolster to the defect (Figure 2), and 1-vicryl interrupted sutures with Surgicel pledget sutures to close the defect, as described by DiBlasio *et al*.^20^ Estimated blood loss was <100ml. The inpatient stay was unremarkable and the man was discharged on postoperative day 4.

**Figure 3:**
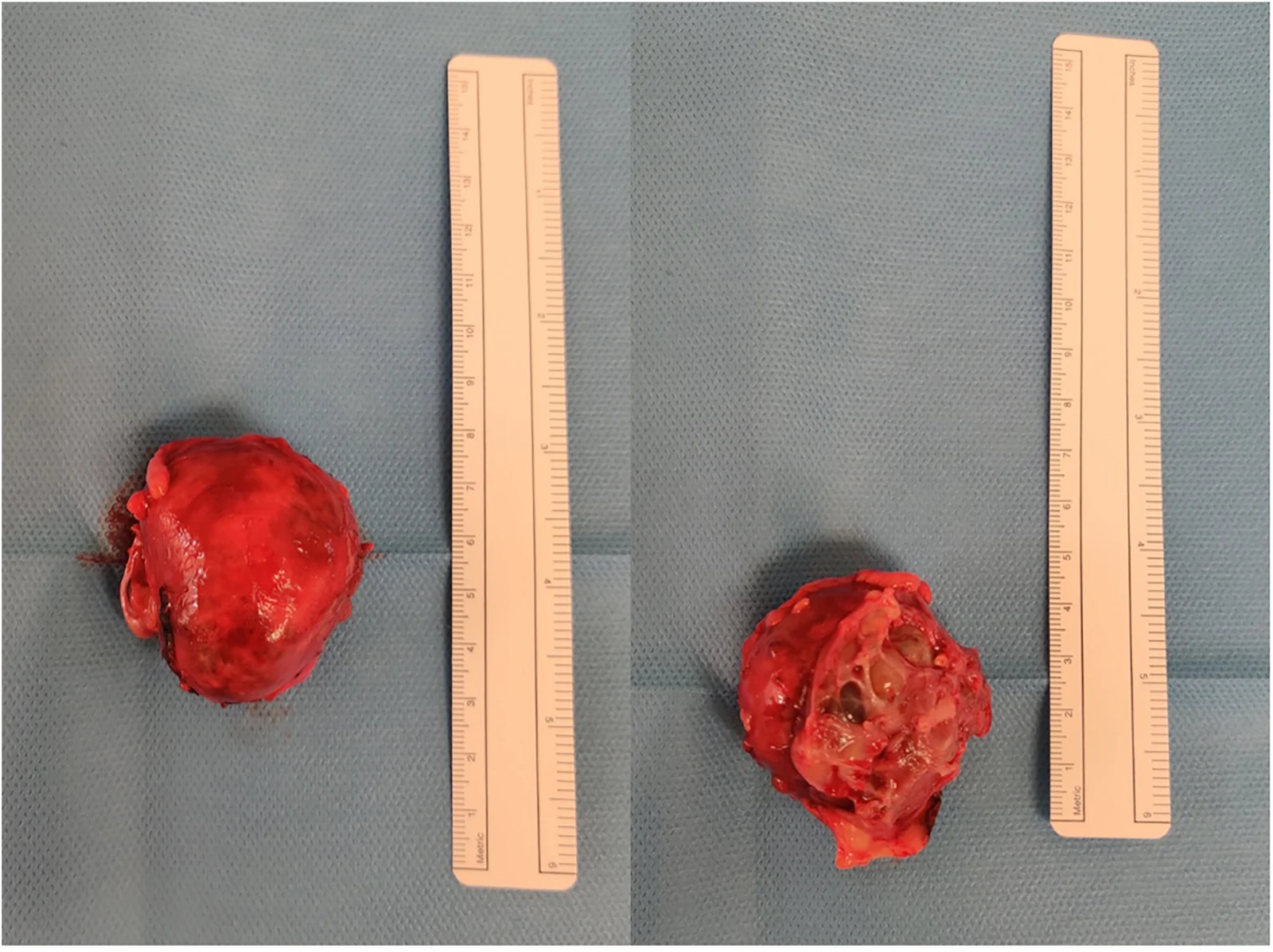
Case 1. Solid renal mass with a margin of normal tissue containing multiple cysts

Final pathology revealed a 4.5cm papillary renal cell carcinoma, International Society of Urological Pathology grade 3, stage pT1b (Figure 3). The patient is recurrence-free at 24 months follow-up, and has a stable eGFR.

### Case 2

A 60-year-old man with ADPKD presented with a slowly enlarging solid mass in the interpolar region of his right kidney. He had CKD related to ADPKD, with an eGFR of 50ml/min. His comorbidities included hypertension, impaired glucose tolerance, gastro-oesophageal reflux disease, primary hyperparathyroidism and a carotid artery aneurysm under surveillance. Biopsy of the mass was technically challenging, and the resulting tissue sample was non-diagnostic. The patient was asymptomatic apart from intermittent mild bilateral flank pain, worse on the left side.

He underwent a zero-ischaemia open right partial nephrectomy via a subcostal incision. Intraoperative ultrasound was utilised to discern the mass in question from the innumerable surrounding cysts. The mass was excised without clamping the renal artery, and post-excision repair was achieved in the same manner as described in Case 1.^20^ His inpatient hospital stay was unremarkable and he was discharged on postoperative day 2. Histology revealed a type 1 papillary renal cell carcinoma, stage pT1a. He is now 5 years post resection with no tumour recurrence.

### Case 3

**Figure 4:**
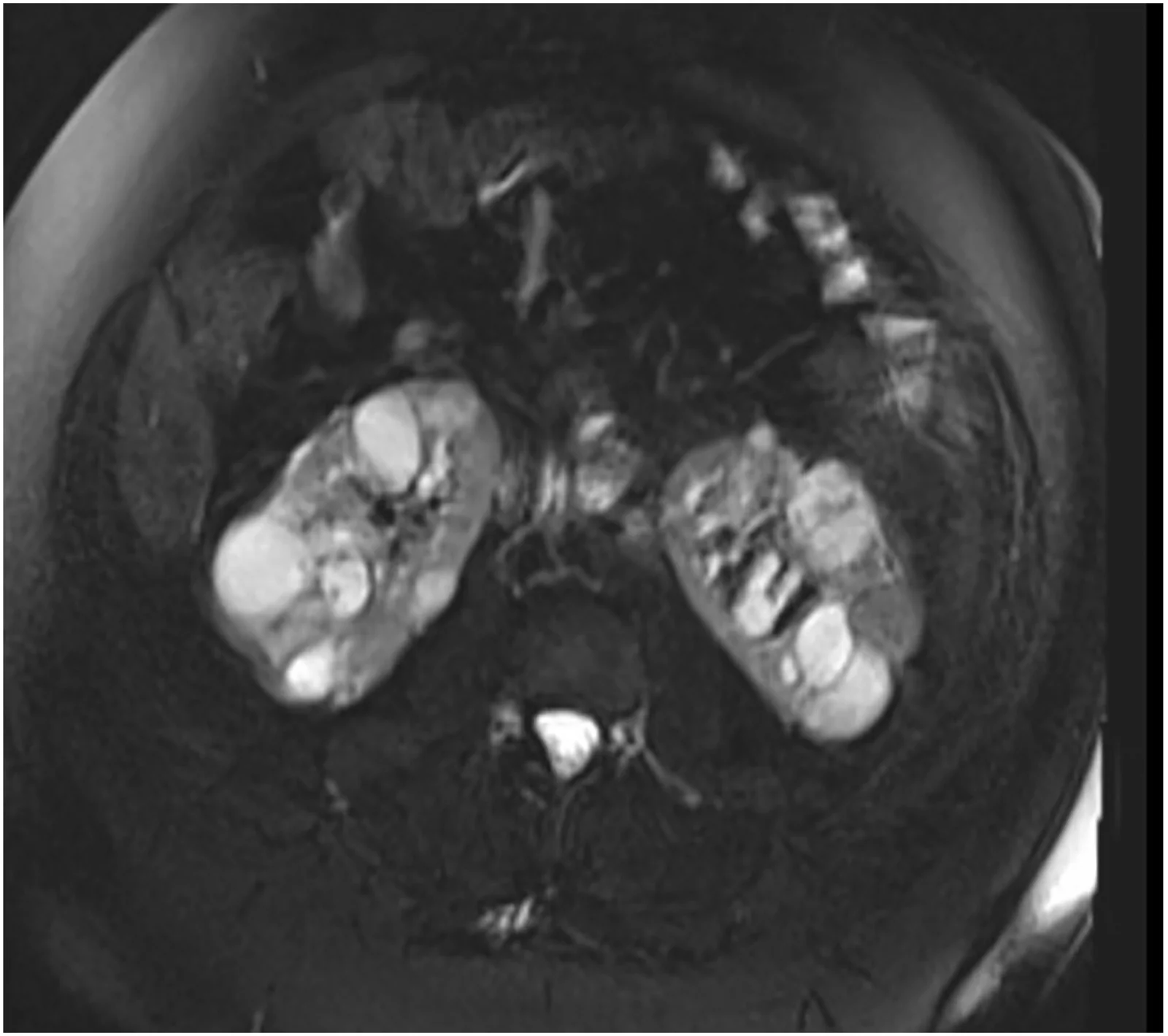
Case 3. Coronal MRI showing a 2.4cm solid enhancing mass in the interpolar region of the left kidney

A 50-year-old woman with ADPKD presented with a solid renal mass, detected incidentally on imaging performed to investigate flank pain during a urinary tract infection. Her last imaging had been 5 years prior to this presentation. Ultrasound revealed a 2.3 × 2.1 × 2.2cm hypoechoic mass in the mid pole of her left kidney, with numerous background renal cysts. She went on to have magnetic resonance imaging (MRI) of her kidneys, which confirmed the presence of a 2.4cm solid enhancing renal mass (Figure 4). Her comorbidities included hypertension and hypothyroidism, and her surgical history included laparoscopic appendectomy, excision of a fibroid cyst from her right breast, laparoscopic cholecystectomy and three Caesarean sections. Her preoperative creatinine was 90μmol/L, resulting in an eGFR of 64ml/min.

She underwent a zero-ischaemia open partial nephrectomy via a left flank incision with the aid of intraoperative ultrasound. Several cysts were decorticated to gain access to the tumour prior to excision. Renorrhaphy was achieved as previously described.^20^ She was discharged on postoperative day 2 with a stable eGFR. Histology revealed a 3cm pT1a renal cell carcinoma, unclassified. She is now 1 year post resection with no recurrence on imaging.

## Discussion

The authors are not aware of any other descriptions of an open partial nephrectomy technique for solid renal masses in patients with ADPKD. A small number of laparoscopic, hand-assisted laparoscopic and robotic partial nephrectomy procedures for solid renal masses in this cohort have been reported. The three cases described here illustrate the technical challenges associated with partial nephrectomy in patients with ADPKD. Common features of all three cases were the use of intraoperative ultrasound to aid in tumour identification, and the need for decortication of surrounding cysts when excising the tumour. Furthermore, ischaemia times can be kept to a minimum in experienced hands, thereby further preserving function in a compromised kidney. Another common feature was the relatively short hospital stay in each case. Histology was malignant in all three patients.

Image-guided tumour ablation could also be considered for small renal masses in patients such as these when a nephron-sparing approach is required. A large meta-analysis found that ablation performed favourably against partial nephrectomy with a lower mean reduction in eGFR post procedure, at the cost of a higher rate of cancer-specific mortality.^21^ Alternatives to surgery including ablation or active surveillance were discussed with all three patients in this series, with each opting for surgical resection after considering all options available to them.

## Conclusion

These cases demonstrate the advantage of pursuing a nephron-sparing approach when a patient is not already on RRT, in order to defer or delay the need to begin dialysis or undergo renal transplantation.
